# The Yellow River is the key corridor for *Tamarix austromongolica* to disperse from Asia inlands to east seashores

**DOI:** 10.1002/ece3.11473

**Published:** 2024-08-07

**Authors:** Hongxiao Yang, Xinwei Liu, Honghao Gan, Jia Sun, Yanxia Pan, Jianmin Chu

**Affiliations:** ^1^ Qingdao Agricultural University Qingdao China; ^2^ Coastal Forestry Research Center of National Forestry and Grassland Administration, Research Institute of Forestry Chinese Academy of Forestry Beijing China; ^3^ Experimental Center of Desert Forestry Chinese Academy of Forestry Dengkou China

**Keywords:** biogeography, dispersal, evolution, riparian plant, *Tamarix* L. genus, the Yellow River

## Abstract

Plants of the *Tamarix* L. genus (Tamaricaceae) mainly occur in arid inlands of Asia, but a few species occur in the coastal areas of China, and the Yellow River may account for this. This study was conducted to elucidate whether and how the Yellow River affects the pattern and development of the *Tamarix* genus, involving two critical species of *Tamarix austromongolica* Nakai and *Tamarix chinensis* Lour. With geographical distribution data, relationships of *T. austromongolica* with the Yellow River and the pertaining watershed were examined using the method of random permutation. The base‐diameter structures of *T. austromongolica* populations were investigated and compared between different riparian lands that suffer discriminative water inundation. The nearest distances from *T. austromongolica* locations to the Yellow River and the pertaining watershed were significantly lower than the theoretical expectations in the condition of random distribution (*p* < .05). In many riparian lands along the Yellow River, wild *T. austromongolica* populations occurred with vigorous juveniles, despite frequent human disturbances. In coastal areas near the present estuary of the river, wild *T. austromongolica* plants were still found. In *T. austromongolica* populations near the Yellow River and sea, the rates of juvenile plants were significantly higher than in other populations situated farther from the river or sea. These findings suggest that the Yellow River can facilitate the eastward dispersal of *Tamarix* plants that reasonably caused the evolution from *T. austromongolica* to *T. chinensis* in ancient coasts in the China east.

## Graphical abstract

This study was conducted to elucidate whether and how the Yellow River affects the pattern and development of the *Tamarix* genus. This work is useful for understanding the ancient evolution from *Tamarix austromongolica* Nakai to *Tamarix chinensis* Lour.

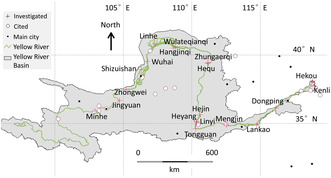

## 1 INTRODUCTION

Most species of the *Tamarix* L. genus (Tamaricaceae) adapt well to subtropical and temperate arid lands, playing an important role in determining ecosystem functions and services (Zhang, 2004; Zhang et al., 2001). The adaptations are inherited from the common ancestors that originated in ancient Mediterranean before the Miocene (Terrones & Juan, 2023; Zhang et al., 2001, 2002). Plants of this genus later dispersed into West and Middle Asia, East Europe, and North Africa, and developed into many lineages and species (Baum, 1978; Villar et al., 2023; Zhang et al., 2002). The Xinjiang Uygur Autonomous Region, China is a secondary distribution center for *Tamarix* to disperse on, and 18 species and one variety exist in China (Liu, 1994; Zhang & Zhang, 1990). Of these species, *Tamarix austromongolica* Nakai and *Tamarix chinensis* Lour. are the critical frontiers to disperse eastward for coastal areas (Liang, Liu, et al., 2019; Liang, Quan, et al., 2019; Wen et al., 2020; Zhang, 2004).

*Tamarix austromongolica* is a widespread shrub species in areas on and near the north‐east Tibetan Plateau, from which the Yellow River originates and flows toward the East Sea. These areas include the Qinghai Province, Gansu Province, Ningxia Hui Autonomous Region, Inner Mongolia Autonomous Region, Shaanxi Province, and Shanxi Province, whereas *T. chinensis* occurs mostly in the eastern provinces of China, such as the Shandong, Hebei, and Jiangsu Provinces (Liang et al., 2018; Wen et al., 2020). *Tamarix austromongolica* was confirmed to be the closest relative to *T. chinensis* because *T. chinensis* was time‐calibrated to evolve from *T. austromongolica* as a young species about 0.69 million years ago in the Quaternary Pleistocene, while *T. austromongolica* originated from another much more ancient *Tamarix* species at least 2.27 million years ago (Liang, Liu, et al., 2019; Liang, Quan, et al., 2019; Sun et al., 2016; Zhang, 2004). About 1.165 million years ago, the ancient Yellow River broke through the Sanmen Gorge now in Henan Province along with the profound uplift of the Tibetan Plateau (Liang et al., 2018; Pan et al., 2005; Wen et al., 2020). After that, some *T. austromongolica* plants were supposed to disperse along the river to eastern coasts, where they were then selected and shaped repeatedly by the coastal environments, at last making the new species of *T. chinensis* (Liang et al., 2018; Zhang, 2004). *Tamarix chinensis* thus adapted to temperate coastal areas relatively better than *T. austromongolica*, and at present it has become a common species in widespread coasts around the Huanghai and Bohai seas (Li et al., 2020; Liu et al., 2023; Sun et al., 2023). In contrast, *T. austromongolica* is routinely absent from these areas, but mainly present in west inlands (Liang et al., 2018).

The Yellow River now enters the Bohai Sea in the Shandong Province (about 37.8° N), whereas thousands of years ago, it used to enter the Huanghai Sea at south ancient sites (about 33° N), now in the Jiangsu Province (Du et al., 2024; Xu et al., 2006; Xue et al., 2004). The current estuary of the Yellow River must not be the initial origin sites of *T. chinensis*, but may carry clues for retrieving the ancient origin of *T. chinensis*, which should be deeply related to hydrological and soil conditions more than climatic ones (Liang et al., 2018). *Tamarix austromongolica* and *T. chinensis* are very similar in morphological traits for their close phylogenetic relationship (Baum, 1978; Liang, Liu, et al., 2019; Liang, Quan, et al., 2019; Zhang, Tan, et al., 2003). Even so, careful taxonomists can clearly distinguish them through the major difference that the inflorescence rachises of *T. austromongolica* are rigid to keep straight or upright, but those of *T. chinensis* are soft to only keep droopy (Zhang & Zhang, 1990). In these contexts, the study was conducted to seek evidences that are meaningful in elucidating the initial origin of *T. chinensis* from *T. austromongolica* and the geographic role of the Yellow River in determining the historical evolution and distribution pattern of *Tamarix* plants in East Asia.

## 2 METHODS

### 2.1 Data collection

**FIGURE 1:**
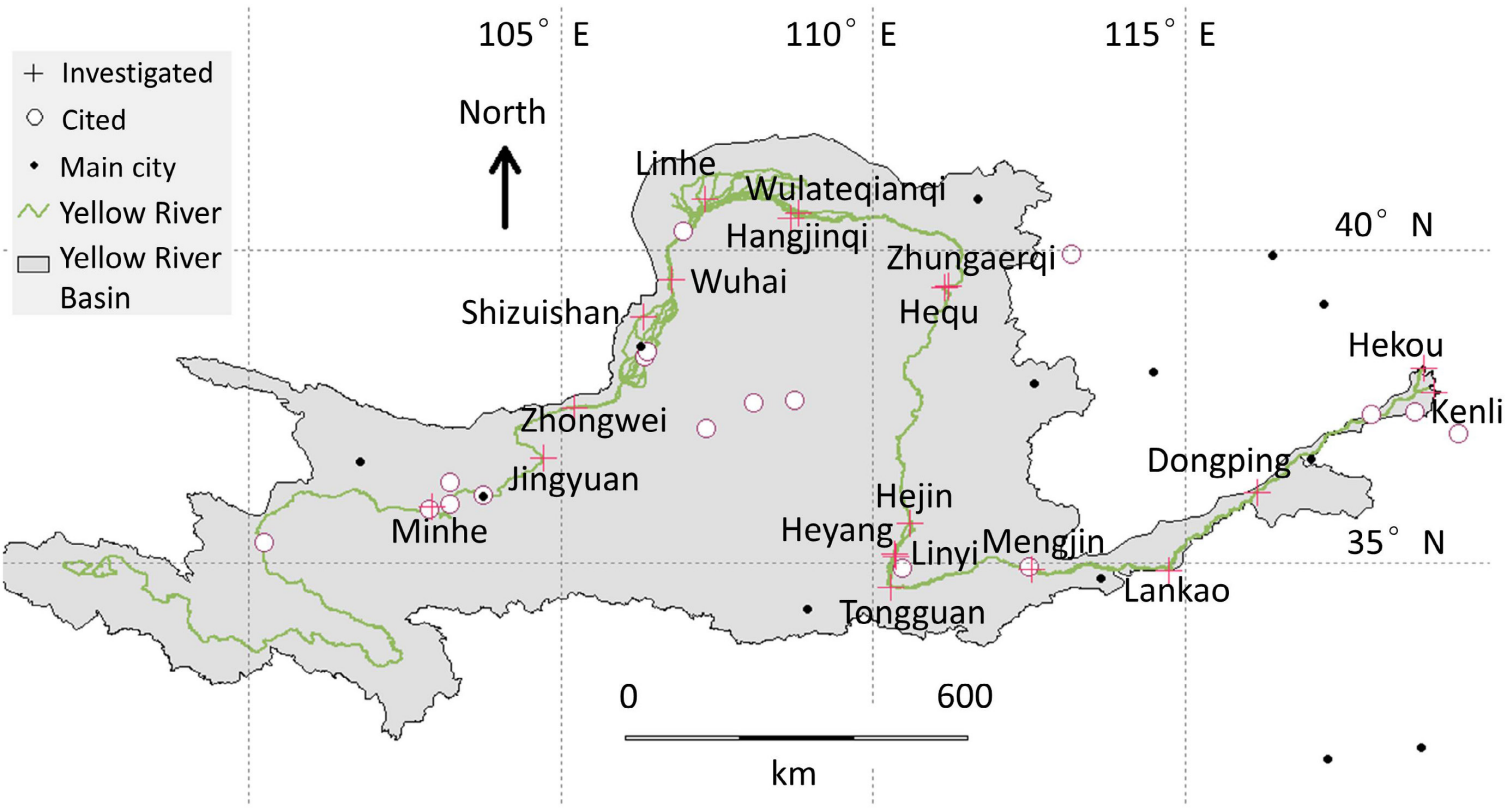
Cited and investigated distribution sites of *Tamarix austromongolica* along with the Yellow River in China. The investigated sites are labeled as in Table 1. The sites in Heyang and Linyi are too near to be clearly discerned, and so are the sites in Zhungaerqi and Hequ.

Occurring sites of *T. austromongolica* were cited from concerned publications (Liang et al., 2018; Wen et al., 2020). Geographic information system (GIS) shape files of the Yellow River and pertaining watershed (Figure 1) were downloaded from the Resources and Environment Scientific Data Center, Chinese Academy of Sciences (www.resdc.cn). In July 2023, wild *T. austromongolica* plants in blossom were investigated on the spot along the banks of the Yellow River, mainly about real occurrences, recruitment state, and human disturbances (Figure 1 and Table 1).

**TABLE 1:** Wild *Tamarix austromongolica* plants investigated along the Yellow River.

Site	Longitude (°)	Latitude (°)	Elevation (m)	Juveniles	Abundance	Terrain	Land use	Disturbance
Minhe	102.9390	35.8939	2230	Occur	Sparse	Slope	Grazing	Light
Jingyuan	104.7255	36.6781	1344	Occur	Sparse	Floodland	Farming	Heavy
Zhongwei	105.2117	37.4848	1173	Occur	Sparse	Floodland	Park	Heavy
Shizuishan	106.3163	38.9440	1066	Occur	Dense	Plain	Park	Light
Wuhai	106.7651	39.5363	1038	Occur	Sparse	Floodland	Park	Heavy
Linhe	107.3149	40.8209	1002	Occur	Sparse	Floodland	Farming	Heavy
Hangjinqi	108.6822	40.5067	993	Occur	Sparse	Floodland	Grazing	Heavy
Wulateqianqi	108.7957	40.5896	982	Occur	Sparse	Floodland	Grazing	Heavy
Zhungaerqi	111.1983	39.4257	961	Occur	Sparse	Floodland	Farming	Heavy
Hequ	111.1349	39.4022	821	Occur	Sparse	Floodland	Park	Heavy
Hejin	110.5922	35.6286	347	Occur	Sparse	Floodland	Grazing	Heavy
Heyang	110.3466	35.1325	329	Occur	Dense	Floodland	Nature reserve	Light
Linyi	110.3595	35.0939	310	Occur	Sparse	Floodland	Farming	Heavy
Tongguan	110.2878	34.6176	290	Occur	Sparse	Floodland	Grazing	Heavy
Mengjin	112.5318	34.8907	99	Occur	Sparse	Floodland	Nature reserve	Light
Lankao	114.7229	34.8838	63	Occur	Sparse	Floodland	Logging	Heavy
Dongping	116.1348	36.1356	46	Occur	Sparse	Floodland	Logging	Heavy
Hekou	118.8111	38.1069	4	Occur	Dense	Beach	Salt airing	Light
Kenli	118.9719	37.7265	2	Occur	Dense	Beach	Nature reserve	Light

In July 2022, flowering *T. austromongolica* plants were identified and investigated in Dengkou (Inner Mongolia Autonomous Region), Yongning (Ningxia Hui Autonomous Region), Yanchi (Ningxia Hui Autonomous Region), and Dingbian (Shaanxi Province), where specialists familiar with forest distribution showed us the major occurring sites. At each site, a random walk route was used to sample 200 *T. austromongolica* plants appearing in sequence on the route, and the measurement item was the base diameter of these plants, including small saplings or juveniles and large adults. In contrast to other sensitive indices, the base‐diameter index is relative steady and easy for measurement and evaluation (Fu et al., 2015; Fu & Shen, 2017). *Tamarix austromongolica* forests were also found in coastal area near the estuary of the Yellow River, but only on a limited scale relative to *T. chinensis* (Wen et al., 2020). In this study, this condition was verified on the spot in August 2022, and the base diameters of *T. austromongolica* plants were investigated at two sites that were in low tidal flat near the sea often with moist salty soil and another evidently higher, drier coastal backland >3 km from the sea. At each site, 20 random quadrats in sizes of 10 m × 10 m were selected to measure and record the base diameters of all *T. austromongolica* plants occurring in the quadrats.

### 2.2 Data analyses

With the cited sites, the dependences of *T. austromongolica* on the Yellow River and related watershed were examined using PASSaGE 2 software, where all the sites were randomly permuted for 499 times, thus calculating theoretical averages of the nearest distances from these sites to the river and watershed and correspondingly the 95% confidence intervals of the theoretical averages on the hypothesized condition of random *T. austromongolica* occurrence, that is, thorough independence of the river and watershed (Rosenberg & Anderson, 2011). The results were additionally verified with the spot investigation data along the river in July 2023.

In Omap Software (www.ovital.com), the nearest distances to the Yellow River were measured from the four investigated sites in Yongning, Dengkou, Yanchi, and Dingbian. Then, the base diameters of *T. austromongolica* plants at these sites were pairwise compared using the Wilcoxon test that works for all data, even not following the statistically normal distribution. Finally, the base diameters of *T. austromongolica* plants near the sea were compared between the two different sites with the *T* test method. These analyses were completed in R4.3.2 software (www.r‐project.org).

## 3 RESULTS

### 3.1 Position relationships with the Yellow River and relevant watershed

The nearest distances from the 38 cited sites to the Yellow River averaged 0.37 degree (Table 2). Provided these sites were random, the distances would average 1.24 degree, with the 95% confidence interval from 1.55 degree to 0.96 degree deriving from the 499 times of random permutations for the 38 sites. Likewise, the nearest distances from the 38 sites to the Yellow River watershed averaged 0.03 degree (Table 2). Provided these sites were random, the distances would average 0.51 degree, with the 95% confidence interval from 0.75 degree to 0.27 degree according to the 499 times of random permutations. These results indicate that the *T. austromongolica* species is very likely to depend on the Yellow River for living and dispersal because the averages of the nearest distances are much lower than the corresponding 95% confidence intervals. This opinion was also confirmed by the spot investigation along the riverbank (Table 1), where *T. austromongolica* plants persisted in recruiting, despite varieties of human disturbances.

**TABLE 2:** Random permutations for evaluating the nearest distances to the Yellow River and pertaining watershed.

Statistic index	To the river	To the watershed
Average of the real nearest distances	0.37	0.03
Average of the theoretical nearest distances	1.24	0.51
Upper 2.5% threshold for the 95% confidence interval	1.55	0.75
Lower 2.5% threshold for the 95% confidence interval	0.96	0.27
Random permutation times	499	499

*Note*: The nearest distance is a composite of longitude and latitude differences from the Yellow River and pertaining watershed, instead of horizontal distances in unit of kilometer.

### 3.2 Base‐diameter structures of *T. austromongolica* populations along the Yellow River and the seashore

**FIGURE 2:**
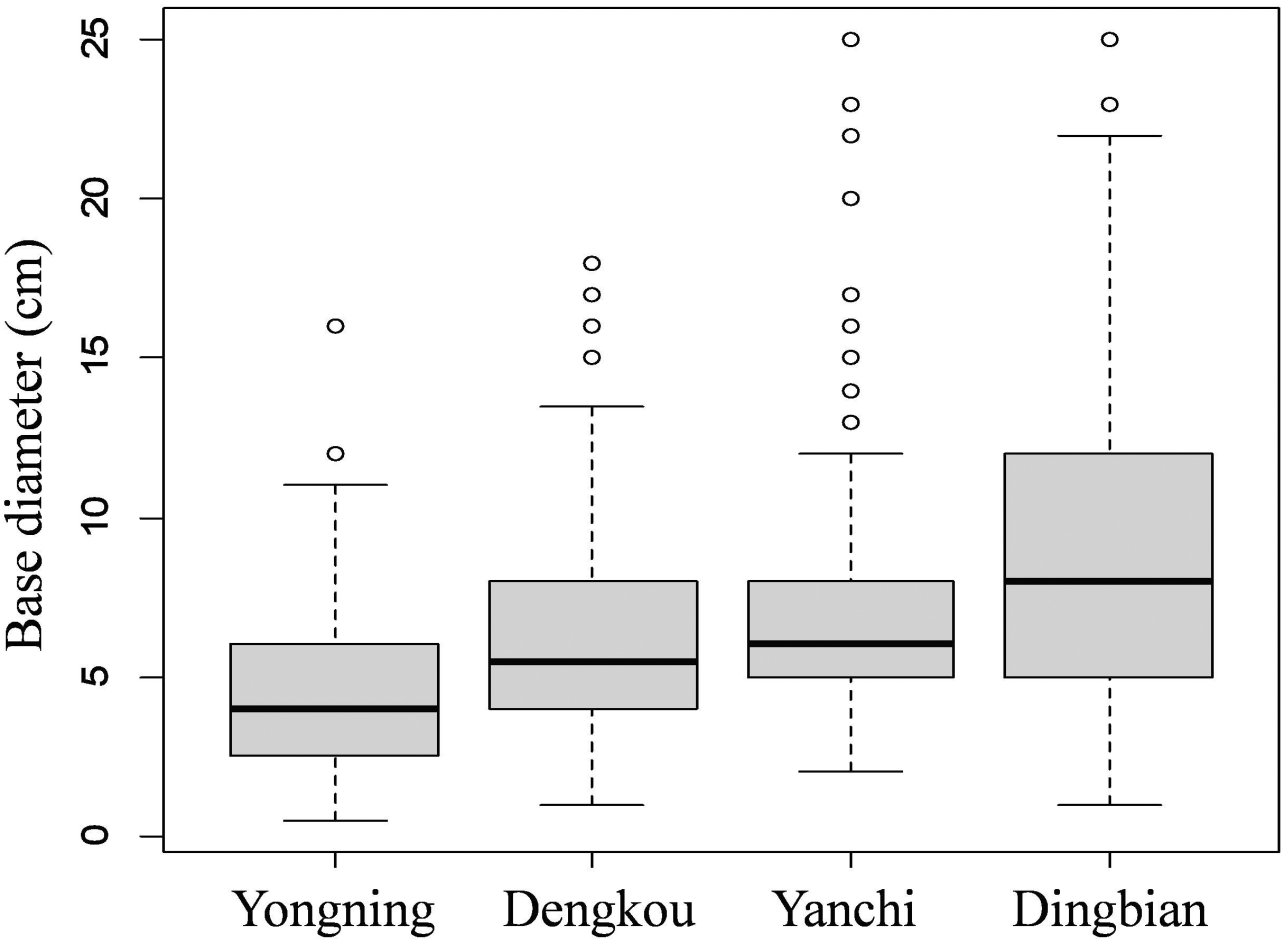
Base‐diameter structures of *Tamarix austromongolica* populations at four sites in the Yellow River watershed.

**FIGURE 3:**
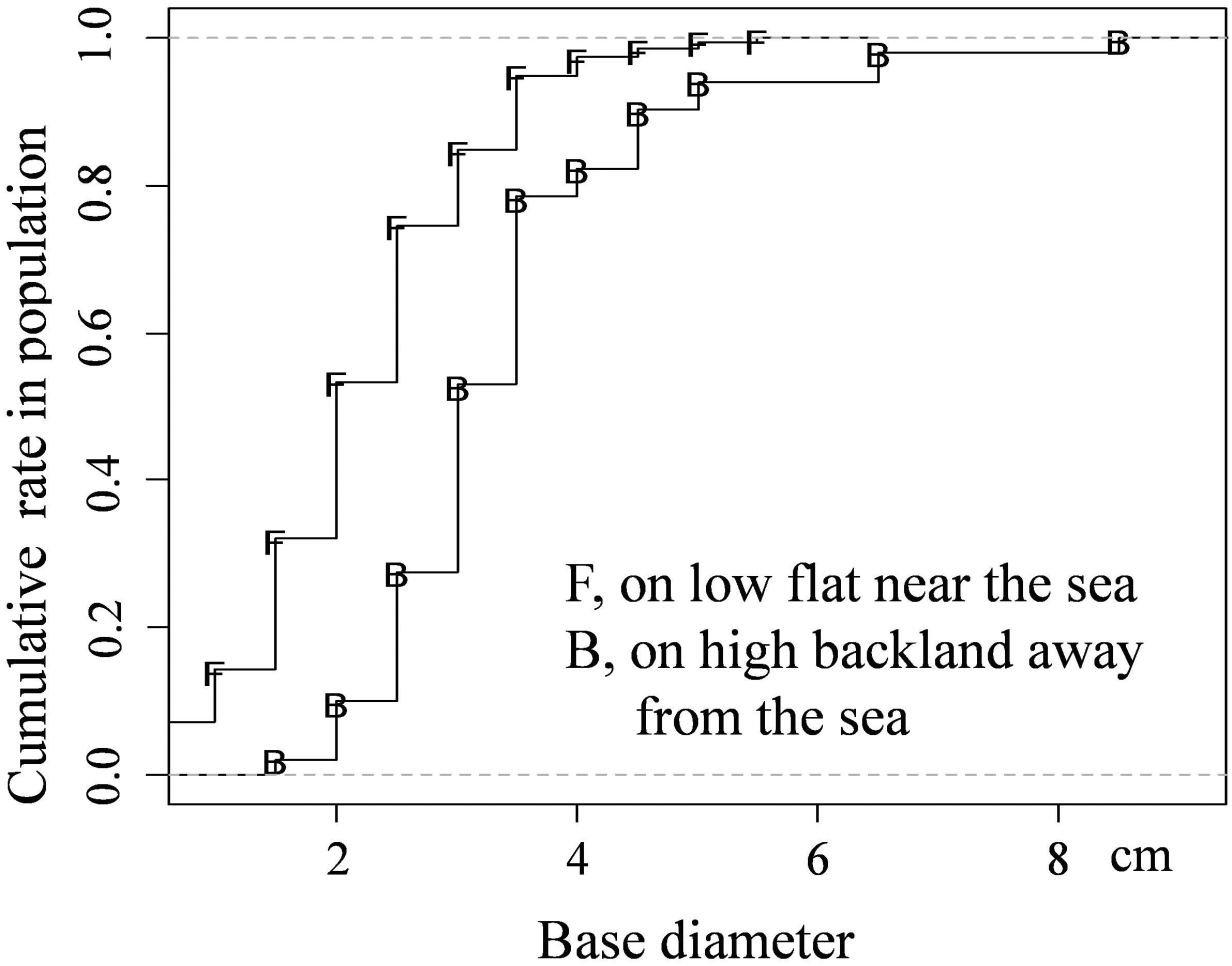
Base‐diameter structures of *Tamarix austromongolica* populations beside the Bohai Sea in different distances.

The nearest distances from the investigated sites (Yongning, 38.3000° N, 106.3424° E; Dengkou, 40.3161° N, 106.9374° E; Yanchi, 37.9416° N, 107.4142° E; Dingbian, 37.5767° N, 108.0914° E) to the Yellow River are 0.1, 7.6, 106.3, and 174.2 km, respectively. The Yongning site is near river water and occasionally flooded in rainy summer. The other sites are lowlands with transient shallow rain accumulation in summer and are not as moist as the Yongning site. At these sites, the *T. austromongolica* populations significantly differ in their base‐diameter structures (Table 3, Figure 2). At the Yongning site, the rate of juveniles is the highest because its average base‐diameter is significantly smaller than those of the three other sites. Likewise, the *T. austromongolica* populations behave differently at the two coastal sites (Figure 3). In general, the young *T. austromongolica* plants are in a higher rate in the low tidal flat than in the high backland, with the base‐diameter averages of 2.22 ± 0.97 cm for the tidal flat and 3.41 ± 1.24 cm for the backland (*p* < .001, *T* test). The results indicate that no matter on low tidal flat or near the Yellow River, *T. austromongolica* population recruits easily with rich saplings or juveniles. That is, moist lands are helpful for *T. austromongolica* plants in occurring.

**TABLE 3:** Comparison of base‐diameters of *Tamarix austromongolica* populations between every two sites.

	Yongning	Dengkou	Yanchi
Dengkou	7.70E‐08**		
Yanchi	6.80E‐15**	1.15E‐2*	
Dingbian	2.00E‐16**	4.90E‐07**	1.60E‐3**

*Note*: The valued significances were derived from the pairwise Wilcoxon test.

*<.05; **<.01.

## 4 DISCUSSION

### 4.1 Dependence of *T. austromongolica* on the Yellow River

The occurrences of *T. austromongolica* populations are closely related to the Yellow River, in spite of the human disturbances, such as farming, grazing, and felling. The Yellow River must help *T. austromongolica* grow. Fruits of *T. austromongolica* are so minute as to adapt to anemochory well, instead of hydrochory, and this species inherits the capability of the *Taxmix* genus to be acclimated to moist, salty riparian lands similar to those in the ancient Mediterranean (Zhang, Pan, & Yin, 2003). Fruits of this species can disperse easily with convenient winds here and there. Provided dropping in moist soils, it is easy for the fruits to start new lives as saplings, juveniles, and adults. The moist lands are common beside the Yellow River. Thus, the major river makes a substantial number of moist habitats to entertain *T. austromongolica* for germinating and growing. This perspective is confirmed again by the further evidence that at the Yongning riparian site and another tidal flat, juveniles of *T. austromongolica* grow in higher rates than at other comparative sites farther from the river or sea.

### 4.2 The role of the Yellow River in *Tamarix* genus evolution

The Yellow River is a key factor for the *Tamarix* genus to disperse further for east areas beside the seas. *Tamarix austromongolica* came into being as a new species about 2.27 million years ago in the ancient reaches of the upper Yellow River, presumably near the current Jishi County, Gansu Province, and later, individuals of this species kept dispersing up and down along the river that were confined to only northwest China (Li et al., 2023; Liang, Liu, et al., 2019; Liang, Quan, et al., 2019; Wen et al., 2020). After the Yellow River broke through the Sanmen Gorge about 1.165 million years ago because of the stepwise uplift of the Tibetan Plateau and the persisting downward river erosion, the river was then conditioned to flow till the east seas (Liang et al., 2018; Liu & Sun, 2007; Pan et al., 2005). As a result, *T. austromongolica* plants could disperse step by step along the river, repeating the life phases of fruit dispersal with winds, fruit dropping in riparian soils, germinating, maturing, and bearing fruits again for dispersal once again. After they arrived in coastal areas, they would be selected by new environment and evolved to adapt more and more to the changed environment, finally bringing forth the new species of *T. chinensis* about 0.69 million years ago (Liang et al., 2018; Liang, Liu, et al., 2019; Liang, Quan, et al., 2019). Taking too many millennia, the *T. chinensis* species now have developed as a widespread dominant species along the lengthy coastlines beside the Bohai and Huanghai seas (Li et al., 2020; Liang, Liu, et al., 2019; Liang, Quan, et al., 2019).

### 4.3 Evidences for the history from *T. austromongolica* to *T. chinensis*

The exceptional occurrence of *T. austromongolica* near the present Yellow River estuary is actually not an impossible odd but a living clue in retrieving the historic evolution of *T. austromongolica* to *T. chinensis* that should have completed in ancient coasts connected to the ancient Yellow River at old estuaries about 0.69 million years ago (Liang, Liu, et al., 2019; Liang, Quan, et al., 2019; Xue et al., 2004). Even now, the *T. austromongolica* plants still disperse along the Yellow River to the present estuary, but because of undeveloped adaptations, they are not enough to challenge the large‐scale dominance of *T. chinensis* across lengthy broad coastal areas (Li et al., 2020; Liu et al., 2023). With massive fruit production and supply, *T. austromongolica* may achieve local dominance near the new estuary of the Yellow River. On this occasion, heredity carried by the *T. austromongolica* plants may be incorporated by *T. chinensis* through smooth interspecific hybridization, which is very common within the *Tamarix* genus, including *T. austromongolica* and *T. chinensis* (Baum, 1978; Sheidai & Koohdar, 2023; Terrones & Juan, 2023). In this case, the arriving *T. austromongolica* are only a complement to *T. chinensis* in further development and evolution, other than a challenge to *T. chinensis* for thriving in much broader coastal areas than now.

## 5 CONCLUSION

The occurrences of *T. austromongolica* are highly dependent on the Yellow River that provides this species with preferred habitats of riparian lands. Along the major river, *T. austromongolica* plants are conditioned to disperse further, and their diminutive haired fruits also facilitate the dispersal through anemochory. The living and dispersing mechanism of *T. austromongolica* must work not only now but also long before the Yellow River met the sea. It is believable that after the Yellow River broke through the Sanmen Gorge and succeeded in meeting the east seas, *T. austromongolica* successfully dispersed into coastal areas and later resulted in the origin of the advanced *T. chinensis*. Now, *T. austromongolica* plants still disperse toward the new estuary of the Yellow River, but they are not sufficient to replace *T. chinensis* as a widespread dominant species along lengthy coastal areas, and can only play as a vivid clue showing the ancient origin of *T. chinensis* from *T. austromongolica*.

## AUTHOR CONTRIBUTIONS

**Hongxiao Yang:** Conceptualization (lead); formal analysis (equal); investigation (equal); writing – original draft (equal). **Xinwei Liu:** Conceptualization (supporting); data curation (equal); investigation (equal); writing – review and editing (equal). **Honghao Gan:** Investigation (equal); resources (equal). **Jia Sun:** Investigation (equal). **Yanxia Pan:** Investigation (equal). **Jianmin Chu:** Conceptualization (equal); data curation (equal); funding acquisition (lead); writing – review and editing (equal).

## CONFLICT OF INTEREST STATEMENT

The authors declare that they have no competing interests.

## Supporting information


Data S1.

## Data Availability

Some data are cited from the website: www.resdc.cn. Our investigated data about the *Tamarix austromongolica* are in the file (ece311473‐sup‐0001‐DataS1.xlsx) where two tables with relevant details are included. One contains the population base‐diameter records along the upper Yellow River, and the other documents the population base‐diameter records on a Bohai Sea coast.
